# Acute Cholecystitis Presenting With Atypical Radiologic or Laboratory Findings: A Case Report

**DOI:** 10.7759/cureus.41217

**Published:** 2023-06-30

**Authors:** Erik Aleman Espino, Mallory Kazaleh, Javier Zaglul, Odalys Frontela

**Affiliations:** 1 Osteopathic Medicine, Nova Southeastern University Dr. Kiran C. Patel College of Osteopathic Medicine, Fort Lauderdale, USA; 2 Internal Medicien, Larkin Community Hospital, Hialeah, USA; 3 Internal Medicine, Larkin Community Hospital, Hialeah, USA

**Keywords:** nonspecific abdominal pain, gangrenous cholecystitis, atypical presentation, clinical case report, acute calculus cholecystitis

## Abstract

Acute cholecystitis is the most common presentation of gallbladder (GB) disease. It has an incidence of around 200,000 cases a year in the United States (US) and affects approximately 20 million individuals in the US. In most cases, it presents with a history of symptomatic gallstones. Initial management includes intravenous hydration and antibiotics, bowel rest, and analgesia. Complicated cases are typically resolved with surgery (laparoscopic cholecystectomy). The pathogenesis of acute cholecystitis is most often explained by obstruction of the cystic duct. Research has shown that there are more contributing factors than just obstruction alone.

We present a case of a 38-year-old Hispanic woman who came to our emergency department with a chief complaint of the anterior chest wall and epigastric pain. Physical examination was remarkable for epigastric tenderness and negative Murphy's sign. She had no fever. Cardiac troponins and electrocardiogram (EKG) were negative. Initial labs showed no sign of infection with white blood cell (WBC) count within the normal range, and only mildly elevated aspartate aminotransferase (AST), alanine transaminase (ALT), and total bilirubin. Follow-up abdominal computerized tomography (CT) scan without contrast and right upper quadrant (RUQ) abdominal ultrasound showed cholelithiasis without evidence of cholecystitis. An hepatobiliary iminodiacetic acid (HIDA) scan on day three of admission revealed an obstruction of the cystic duct. The patient was scheduled for laparoscopic cholecystectomy with an intraoperative cholangiogram. The surgery was uneventful; it was remarkable for a very distended, inflamed, and edematous GB, which had to be decompressed with a lap needle for removal.

It is evident that acute cholecystitis may not always present with the classic diagnostic criteria, including laboratory results (leukocytosis, elevated C-reactive protein) and physical exam findings (fever, RUQ pain, and + Murphy’s sign). However, a thorough work-up can be just as effective in diagnosis.

## Introduction

Acute cholecystitis is the most common presentation of gallbladder (GB) disease. It has an incidence of around 200,000 cases annually in the United States and affects approximately 20 million individuals [1]. In most cases, it presents with a history of symptomatic gallstones. Initial management includes intravenous hydration and antibiotics, bowel rest, and analgesia. Complicated cases are typically resolved with surgery (laparoscopic cholecystectomy) [2,3].

The pathogenesis of acute cholecystitis is most often explained by obstruction of the cystic duct [2]. However, research has shown that there are more contributing factors than just obstruction alone. Some studies describe the need for an additional irritant as a requirement for cholecystitis to occur. One commonly described irritant in experimental models is lysolecithin, which is produced from a normal constituent of bile: lecithin. This theory is supported by the presence of phospholipase A in the GB. Phospholipase A catalyzes the reaction and may be released after trauma from a gallstone impaction. Inflammation then leads to the release of more inflammatory mediators [4]. Another proposed theory is an infective insult; however, records show that not every patient with cholecystitis has infected bile. When an infection is present, it is usually due to *Escherichia coli*, Enterococcus, Klebsiella, or Enterobacter. Cholecystitis histologic changes range from mild findings, such as edema and acute inflammation, to more severe changes, including gangrene and necrosis [2,3].

Acute cholecystitis typically presents with right upper quadrant (RUQ) pain or epigastric pain that often radiates to the right shoulder or the back. The pain is usually associated with onset after ingesting fatty meals. It usually lasts a prolonged time (four to six hours) and stays steady at high intensity. Some associated symptoms include nausea, vomiting, fever, and anorexia [2,3]. Physical examination typically shows an ill-appearing patient lying still in bed since the pain is visceral. Patients may show guarding on the abdominal exam, along with pain during inspiration and holding while the clinician palpates the right subcostal area (+ Murphy’s sign). Labs usually show leukocytosis with elevated bands. Since the obstruction is limited to the cystic duct, total serum bilirubin and alkaline phosphatase (ALKP) elevation are uncommon. It has been reported that a small elevation in liver enzymes (alanine transaminase (ALT), aspartate aminotransferase (AST)) and amylase can be present in a minority of cases [2].

The presence of gallstones associated with RUQ pain and fever highly supports the diagnosis of cholecystitis. Abdominal ultrasound is a wildly used tool with a sensitivity and specificity of 84% and 99%, respectively. Sonographic findings show GB wall thickening along with pericholecystic fluid and edema (double wall sign). A sonographic Murphy’s sign can also aid in the diagnosis [2,5].

A hepatobiliary iminodiacetic acid (HIDA) scan is the next best step if the diagnosis remains inconclusive after ultrasonography. This shows a physiological picture of the GB. In an average healthy patient, visualization of contrast occurs within 30 to 60 minutes after administration. A delay in the visualization of the GB after 60 minutes or after delayed imaging (3 to 4 hours) supports a diagnosis of acute cholecystitis in the correct clinical setting [3,6].

A computerized tomography (CT) scan of the abdomen, which has a high sensitivity (94%) but low specificity (59%) is not usually indicated for the diagnosis of acute cholecystitis, may be ordered to rule out other conditions before a final diagnosis is made. Findings include wall edema, high-attenuation bile, and pericholecystic stranding and fluids [3].

## Case presentation

A 48-year-old female with a past medical history of gastroesophageal disease (GERD) and obesity presented to the emergency room via private vehicle complaining of chest and abdominal pain. The pain started the day before after she had lunch and continued bothering her throughout the day. The pain was described as sharp and located on the anterior chest wall without significant radiation. It was associated with nausea and four episodes of non-bloody emesis. The patient denied alcohol, tobacco, and illicit drug usage, as well as the presence of other significant symptoms.

**Figure 1:**
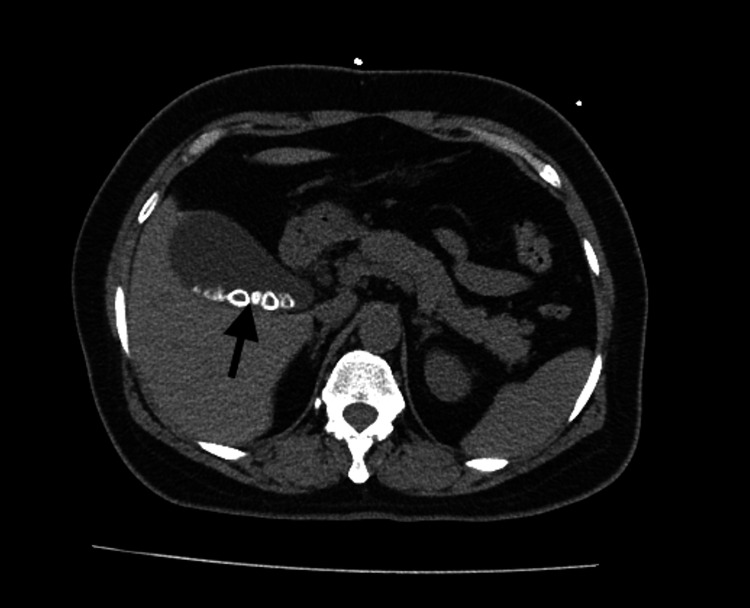
CT abdomen and pelvis without contrast. Significant for a non-distended GB, numerous radiopaque gallstones (black arrow), no wall thickening or pericholecystic fluid, and no intra- or extra-hepatic biliary duct dilation. GB, gallbladder

On examination, the patient was alert, oriented, and in moderate distress, with vitals within normal range. Physical exam was remarkable for tenderness at the epigastric region with no guarding or rebound tenderness. Murphy's sign was negative. The patient had features of obesity. Lab work was significant for ALKP: 102, ALT: 52, AST: 52, and total bilirubin (TBILI): 2.6. Cardiac troponins and electrocardiogram (EKG) were negative. Abdominal CT (Figure 1) showed cholelithiasis without cholecystitis and a lobulated appearance of the uterus.

**Figure 2:**
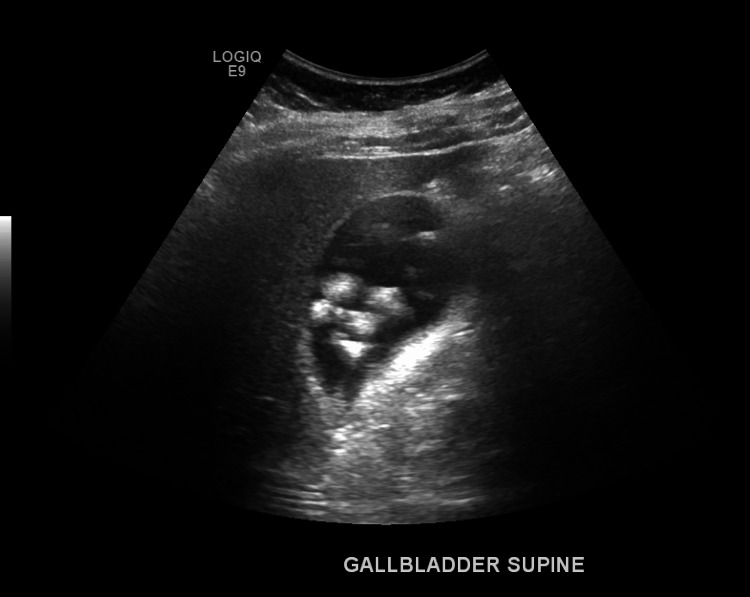
RUQ abdominal ultrasound revealed a non-distended GB with multiple shadowing gallstones. The GB wall measured 3 mm with no thickening, no wall hyperemia, and no pericholecystic fluid. RUQ, right upper quadrant; GB, gallbladder

On day one after admission, the patient continued to display tenderness in the epigastric region without guarding or rebound tenderness. Murphy's sign was still negative. The patient received morphine 2 mg for pain, but she continued to report persistent mild pain. Abdominal ultrasound RUQ (Figure 2) revealed cholelithiasis without sonographic evidence of acute cholecystitis. The gastroenterology (GI) specialists were consulted and brought onto the team. Due to suspicion that the patient was having a myocardial infarction and in accordance with hospital protocol, she was started on nitroglycerin 2% BID, Lovenox 40 mg SQ QD, Metoprolol 25 mg PO BID, aspirin 81 mg, and Lisinopril 5 mg PO QD until she got clearance from the cardiology team. The echocardiogram ordered on admission was reviewed and showed preserved EF with no valvular abnormalities.

Abdominal ultrasound report - LIVER: The liver measures 13.8 cm long. Unremarkable echogenicity and echotexture; smooth surface contours. No focal lesions were identified. BILIARY TREE: No intrahepatic or extrahepatic biliary dilatation. The common bile duct was non-dilated and measured 4 mm. GB: Non-distended; multiple shadowing gallstones. The GB wall was not thickened and measured 3 mm. No wall hyperemia. No pericholecystic fluid. The sonographic Murphy's sign was negative.

On day two of admission, the patient was feeling much better. The pain had improved, although she did report some RUQ tenderness along with epigastric tenderness. She denied current chest pain, nausea, or vomiting.

**Figure 3:**
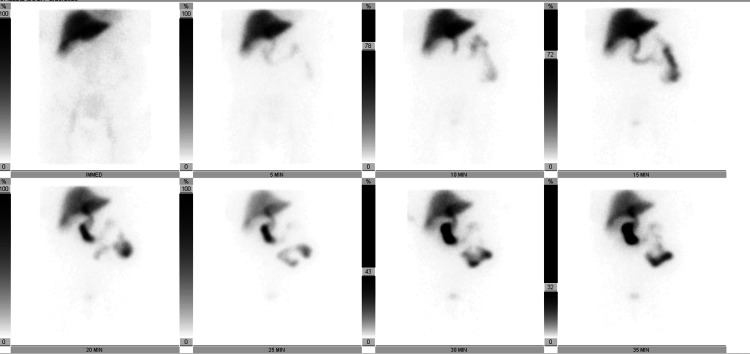
Nuclear medicine hepatobiliary scan. Radiotracer activity was not seen within the gallbladder throughout the duration of the exam, including four-hour delayed imaging.

A follow-up HIDA scan (Figure 3) on day three showed prompt uptake and homogeneous activity in the liver. Excretion of radiotracer activity from the liver was normal. There was a prompt activity in the common bile duct by five minutes. Radiotracer activity was not seen within the GB throughout the duration of the exam, including at four-hour delayed imaging, confirming cystic duct obstruction.

The patient was scheduled for laparoscopic cholecystectomy with intraoperative cholangiogram under the diagnosis of acute fibrinous cholecystitis. The surgery was uneventful and it was remarkable for a very distended, inflamed, and edematous GB. Once this was noticed, the GB had to be decompressed with lap needle for removal. Next, the GB was retracted over the dome of the liver and cephalo-lateral. The cystic duct was then clipped at the GB junction, and after a small ductotomy, the cholangiogram was performed. The cystic artery was also dissected and clipped proximally and distally. The GB was removed from the liver bed using an electrocautery hook, which allowed the surgeon to place it into an endocatch and remove it through the transumbilical port. The patient tolerated the procedure well. The patient was extubated in the operating room without event and transferred to the recovery room in stable condition.

On postoperative day two, the patient was seen and evaluated by the medical team. The patient reported no complaints and was resting in her bed comfortably. The patient reported she was passing gas and walking with assistance. The patient was successfully advanced to a mechanical soft diet and was recommended for discharge.

## Discussion

GB disease is common in Hispanic, American, and Native American populations. Acute cholecystitis spans the following patient populations: 35.2% Latinos, 23.3% Japanese, 19.4% Whites, 18.4% African Americans, and 3.8% Native Hawaiians. Due to the wide variety of symptoms, physicians must have a high index of suspicion when evaluating patients presenting with abdominal or chest pain [7,8]. A complete history and physical exam should always be done on all patients followed by non-invasive testing. A positive Murphy’s sign on physical exam, RUQ pain, fever, and leukocytosis indicate cholecystitis. However, radiological imaging that shows wall thickening and edema, a positive sonographic Murphy’s sign, or failure of the GB to light up on the HIDA scan is required to establish the diagnosis [1,2]. In the case we presented, the patient did not have the most common signs and symptoms of GB disease. The medical team had to rely on additional tests to arrive at the final diagnosis and treatment. Diagnosis and treatment delays may lead to more severe conditions like gangrenous cholecystitis, increasing complications, and mortality risks. 

Studies have shown that cholecystitis may present asymptomatically in the geriatric population. In their study “Emergency Department Evaluation of Geriatric Patients with Acute Cholecystitis,” Parker et al. concluded that patients greater than 65 years old display an increased incidence of atypical presentations [9]. This middle-aged patient presenting with atypical symptoms makes this case interesting [9]. Laboratory results (leukocytosis, C-reactive protein, GGT) and physical exam findings (fever, RUQ pain, and + Murphy’s sign) are helpful in guiding the diagnosis. Still, they are not always reliable, as shown by our patient. Additional challenges we encountered were the negative CT/ultrasound findings and the atypical pain pattern, which delayed the diagnosis [3,10].

Ultimately, we relied on the HIDA scan for diagnosis. HIDA’s sensitivity and specificity are approximately 90-97% and 71-90%, respectively [8]. In Solomon et al., it was reported that the use of morphine, which was administrated to our patient, helps to increase the sphincter of Oddi pressure [3]. This, in turn, causes a larger pressure gradient, allowing the radiotracer to move into the cystic duct. It is hypothesized that the use of morphine could be particularly useful in critically ill patients whose HIDA scans are associated with false positives [11]. Other causes of false positives include cystic duct obstruction due to a stone or incomplete cystic duct obstruction. The definitive treatment of acute cholecystitis is cholecystectomy. Poor surgical candidates may be treated with antibiotics and GB drainage procedures [3]. Our patient was taken into surgery following guidelines and a laparoscopic cholecystectomy with intraoperative cholangiogram was performed. During surgery, it was noted that the GB was already inflamed and required decompression to be extracted. It was possibly on its way to turning into necrotizing cholangitis.

## Conclusions

It is evident that acute cholecystitis may not always present with the classic diagnostic criteria, including laboratory results (leukocytosis, C-reactive protein) and physical exam findings (fever, RUQ pain, and + Murphy’s sign). However, a thorough work-up can be just as effective in diagnosis. Early detection and treatment of acute cholecystitis are vital due to the possibility of complications if left undiagnosed. In our patient, a prompt and excellent work-up prevented the development of gangrenous cholecystitis, a potentially life-threatening complication. By having a high index of suspicion for cholecystitis with each complaint of abdominal pain, we can improve patient outcomes and prevent mortality.
